# Potential Mechanisms of Chlorogenic Acid in Cognitive Impairment: A Comprehensive Review

**DOI:** 10.3390/molecules31213800

**Published:** 2026-10-22

**Authors:** Yuxuan Liu, Shijun Wang

**Affiliations:** 1 College of Traditional Chinese Medicine, Shandong University of Traditional Chinese Medicine, Jinan 250300, China; 2024120632@sdutcm.edu.cn; 2 Shandong Co-Innovation Center of Classic Traditional Chinese Medicine Formula, Shandong University of Traditional Chinese Medicine, Jinan 250300, China

**Keywords:** chlorogenic acid, cognitive impairment, neuroprotection, gut–brain axis, neuroinflammation, oxidative stress, autophagy, apoptosis, amyloid-β

## Abstract

Cognitive impairment (CI) is a clinical syndrome primarily affecting cognitive functions such as learning, memory, perception, and problem-solving. Its causes are heterogeneous, including neurological, psychiatric, metabolic, vascular, toxicological, and age-related factors. Currently, no specific and effective pharmacological therapies are available for CI in clinical practice. As one of the most abundant dietary polyphenols, chlorogenic acid (CGA) displays diverse biological effects and has recently garnered growing interest within cognitive neuroscience. This review summarizes the mechanisms by which CGA ameliorates cognitive impairment, encompassing neuroprotection, modulation of the gut–brain axis, inhibition of neuroinflammation, inhibition of oxidative stress, promotion of autophagy, inhibition of apoptosis, and suppression of amyloid-β (Aβ) formation. The signaling pathways implicated include activation of the PKC, Nrf2/PPAR and Nrf2/ARE pathways, as well as inhibition of the TLR4/NF-κB, mTOR/TFEB, p38 MAPK, and JNK/c-Jun pathways. This article provides an overview of current advancements in CGA, addressing its chemical attributes, dietary origins, drug metabolism, and the mechanistic basis for its efficacy in neurodegenerative diseases.

## 1. Introduction

Cognitive impairment (CI) constitutes a clinical syndrome that chiefly impacts higher-order cognitive functions, such as learning, memory, perception, and reasoning [1]. It constitutes a complex syndrome with a broad spectrum of manifestations, ranging in severity from mild cognitive decline to dementia [2]. Studies have identified cognitive decline as one of the most common age-related health issues among older adults [3], with its prevalence increasing with advancing age and lower educational attainment [4], Among adults aged 60 and older, the estimated prevalence of mild cognitive impairment ranges from 6.7% to 25.2% [5]. The consequences of CI include reduced physical activity, physical disability [6], loss of social engagement [7], an elevated risk of developing dementia [6] and increased healthcare expenditures [8]. The currently recognized pathogenic mechanisms encompass impaired neuroprotective mechanisms [9], alterations in gut microbiota homeostasis and their metabolites [10], chronic neuroinflammation [11], oxidative stress and autophagic dysregulation [12,13] and direct neurotoxicity induced by amyloid-β (Aβ) plaque deposition [14,15]. However, no effective pharmacological treatment is currently available for mild cognitive impairment [16] or for vascular cognitive impairment and dementia (VCID) [17]. And for Alzheimer’s disease (AD), available therapies (e.g., Acetylcholinesterase inhibitors, memantine, and anti-amyloid antibodies) offer only limited symptomatic or disease-modifying benefit [18], making the prevention and treatment of CI a prominent research priority [19].

Previous studies have demonstrated that moderate intake of dietary polyphenols can ameliorate cognitive impairment [20]. Chlorogenic acid (CGA) ranks as the most abundant polyphenol found in human dietary intake, with daily intake reaching the milligram-to-gram level [21]. Over the past few years, CGA has been acknowledged for its ability to protect neurons and boost cognitive function, and animal studies have demonstrated its efficacy in treating cognitive impairment [22]. Its therapeutic potential is garnering increasing attention in the field of cognitive research. This review synthesizes the literature from the past thirty-five years concerning CGA in the treatment of cognitive impairment, and focus on age-related CI in older adults, which spans the continuum from normal age-related cognitive decline to mild cognitive impairment (MCI) and Alzheimer’s disease (AD) dementia, providing an updated and comprehensive evaluation to inform future clinical investigations of CGA for neurodegenerative diseases.

## 2. Literature Search and Selection

We searched the PubMed database from 1 November 1992 to 2 July 2026 (https://pubmed.ncbi.nlm.nih.gov/ (accessed on 13 August 2026)). The search terms combined the subject terms and free words, including chlorogenic acid, 5-caffeoylquinic acid, as well as cognitive impairment, cognitive decline, etc. The final search date was 13 August 2026. After removing duplicates and screening 249,900 records, 107 studies ultimately met the inclusion criteria. We included original in vivo, in vitro and randomized controlled trials, requiring chlorogenic acid as the main intervention and reporting cognitive-related outcomes; exclude preprints, conference abstracts, case reports, duplicate literature, and retractions. We extracted species/cells, models, doses, administration routes, treatment courses, controls, outcomes and mechanisms. Since this study was a narrative review, no formal bias risk assessment tool was used; instead, the quality of evidence was narratively evaluated based on elements such as study design, sample size, randomization/blinding, model validity, dose correlation, objectivity of cognitive results, and directness of evidence. Liver and intestinal studies were included in the main review because they are related to the gut–brain axis; studies not involving cognitive results were identified separately by cell type and clearly labeled as indirect evidence. The research results were narratively synthesized by mechanism. We used narrative synthesis and summarized the findings by mechanism.

## 3. Chemical Properties of Chlorogenic Acid

**Figure 1:**
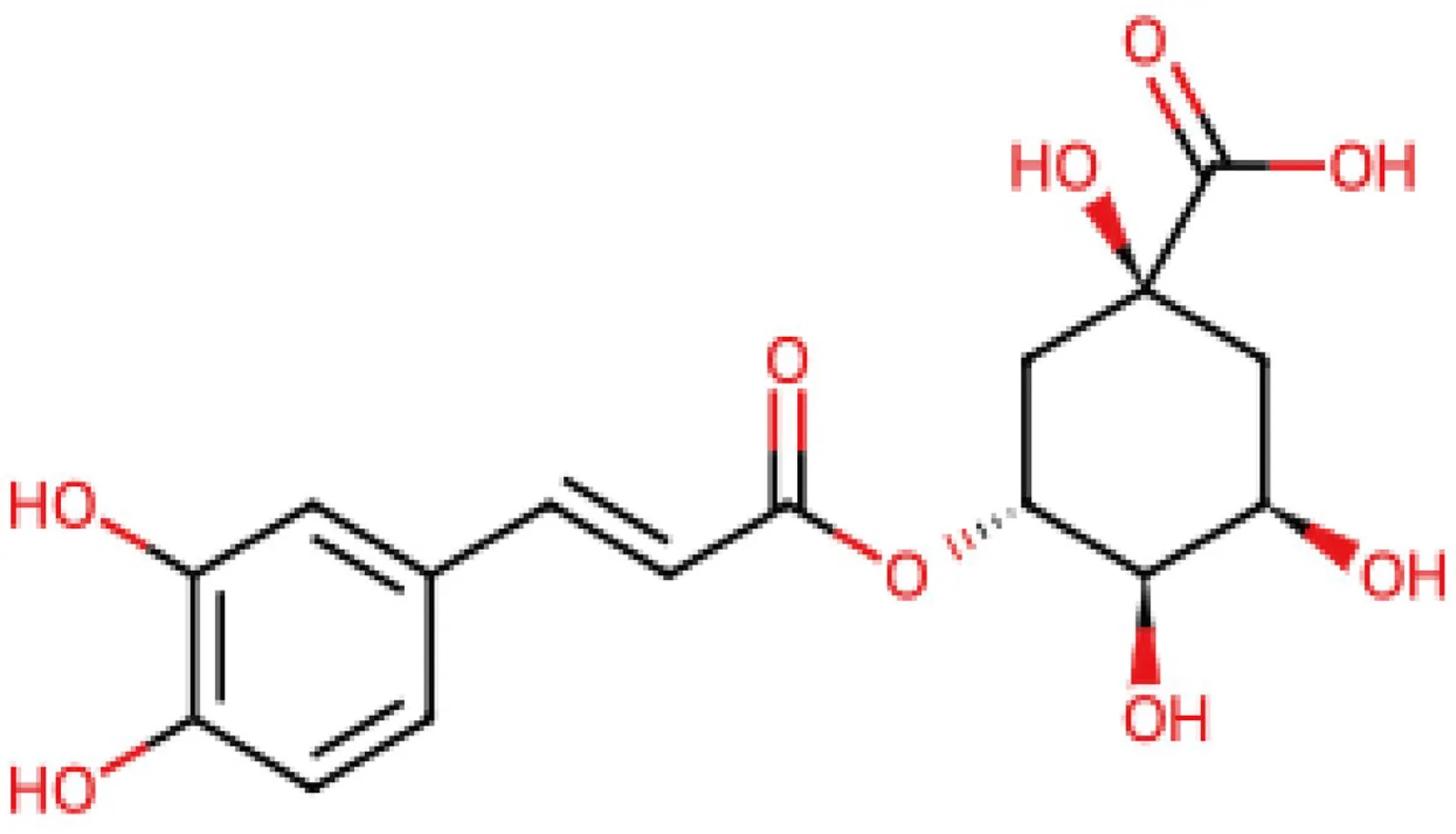
Molecular structure of chlorogenic acid.

Chlorogenic acid (CGA), or 5-caffeoylquinic acid (5-CQA), is a naturally derived phenolic acid. As a phenolic acid bearing an ortho-hydroxyl group, CGA is formed through the esterification of caffeic acid and quinic acid [23,24]. In the Traditional Chinese Medicine Systems Pharmacology (TCMSP) database, this substance is registered under the molecular identifier MOL001955. CGA appears as a white to pale yellow-beige powder with a density of 1.7 ± 0.1 g/cm^3^. Its molecular formula is C_16_H_18_O_9_, with a molecular weight of 354.31 g/mol and a melting point of approximately 205–209 °C. CGA is soluble in water, with a solubility of 40 mg/mL at 25 °C. The molecular structure of CGA is shown in Figure 1.

## 4. Sources of Chlorogenic Acid

Chlorogenic acid cannot be synthesized de novo by animals or humans and must therefore be obtained from dietary sources. CGA is widely distributed in food plants, including sweet potato [25], potato [26], Asian pear [27], apple [28], and coffee [29]. Additionally, CGA is abundantly present in numerous traditional Chinese medicines, including *Lonicera japonica* (Jinyinhua) [30], *Chrysanthemum morifolium* (Juhua) [31], *Phellodendron amurense* (Huangbo) [32], *Cichorium intybus root* (Jujuigen) [33], *Bretschneidera sinensis* (Bolaishu) [34], *Foeniculum vulgare* (Huixiang) [35], *Eucommia ulmoides* (Duzhong) [36], *Schisandra chinensis* (Wuweizi) [37], *Acanthopanax senticosus* (Ciwujia) [38], *Aralia nudicaulis* L. [39], and *Sanghuangporus baumii* (Pilát) [40]. The CGA content varies among these sources (Table 1).

## 5. Pharmacokinetics of Chlorogenic Acid

### 5.1. Safety

No adverse events were reported in the randomized clinical trials included in a systematic review and meta-analysis of CGA supplementation [41]. These data suggest that CGA is generally well tolerated in humans, although the evidence base remains limited and heterogeneous [41]. Kitts and Wijewickreme further confirmed that mice exposed to dietary chlorogenic acid (0.2%, 2000 ppm) for 10 weeks exhibited no toxic reactions, with no effects on body, liver, or intestinal weights [42].

### 5.2. Pharmacokinetics

**Figure 2:**
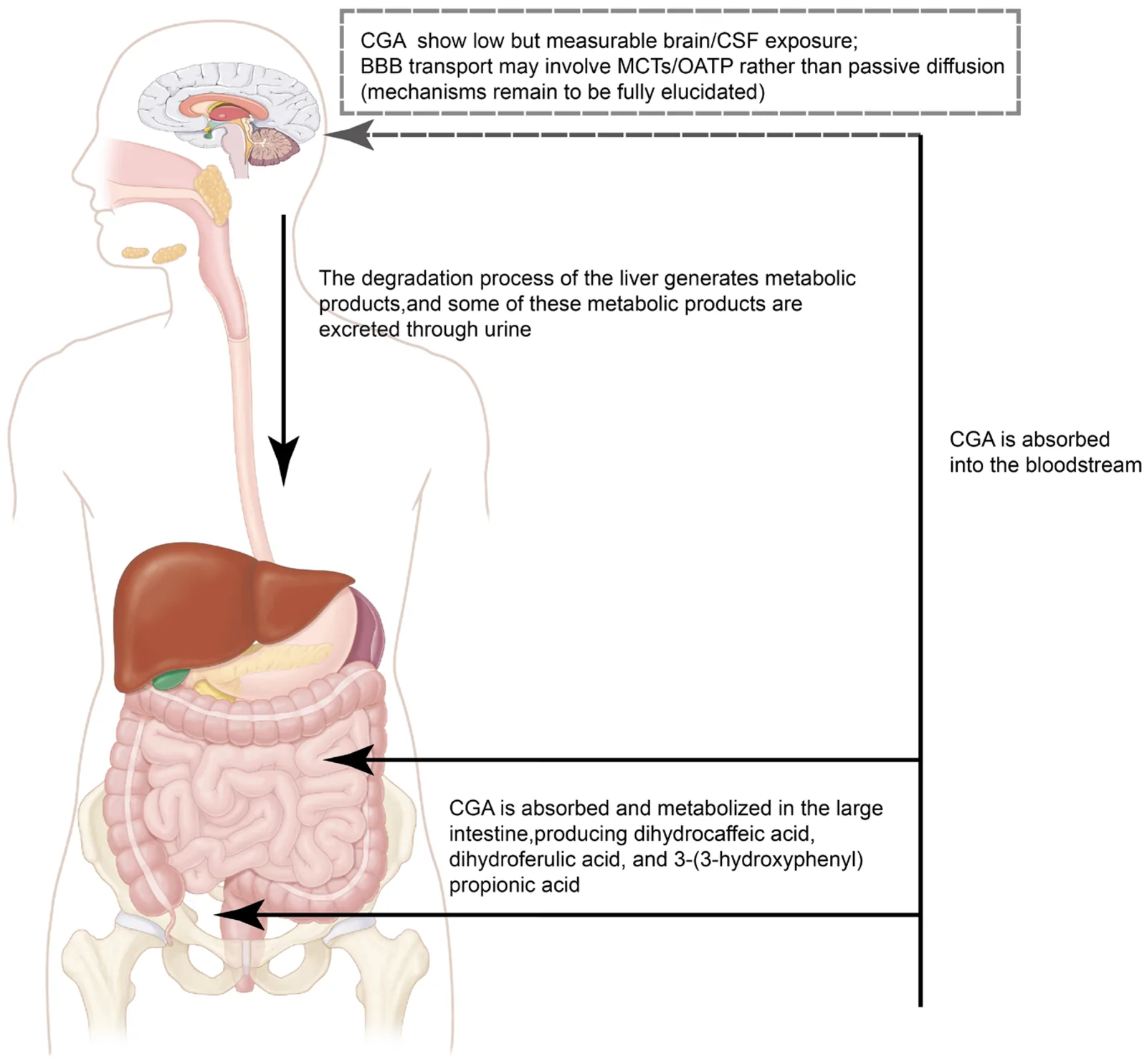
Pharmacokinetics of chlorogenic acid. Symbols and abbreviations: The established observational results are represented by solid-line arrows, while the proposed transmission mechanism is indicated by dashed-line arrows and dashed-line box. Image provided by National Institute of Diabetes and Digestive and Kidney Diseases, National Institutes of Health.

CGA has low oral bioavailability. In rats, gastric administration of 2.25 μmol CGA yielded only 0.12 μmol/L intact CGA in the portal vein and 0.04 μmol/L in the abdominal aorta at 5 min [43]. In situ intestinal perfusion showed that net CGA absorption was 8% of the perfused flux, intact CGA was not detected in plasma or bile, and biliary excretion was <0.4%, suggesting limited enterohepatic recirculation [44]. Upon entering the large intestine, CGA is hydrolyzed by the microbial community [45,46]. Most CGA reaches the colon, where microbial esterases hydrolyze it to caffeic acid and quinic acid [45,46]. Human faecal extract converted 8 μg of 21 μg CGA to caffeic acid within 2 h [45], and *E. coli* hydrolyzed about 80% of CGA within 1 h [46]. CGA undergoes reduction, methylation, sulfation, and glucuronidation [47] to yield 11 key metabolites, with the principal products being dihydrocaffeic acid, dihydroferulic acid, and 3-(3-hydroxyphenyl) propionic acid [48]. Furthermore, the absorption and excretion of CGA can be inhibited through interactions with Streptococcus salivarius and other microbial components [49]. Although CGA and its metabolites can be detected in the brain or cerebrospinal fluid after administration, their blood–brain barrier (BBB) permeability is generally low, and the transport mechanisms—rather than simple passive diffusion—remain to be fully elucidated. This limited but measurable brain exposure may nonetheless contribute to the neurotrophic and neuroprotective effects observed in preclinical models [50]. More importantly, the structural similarity of CGA to certain phenolic acids suggests that it may undergo trans-endothelial transport via monocarboxylate transporters (MCTs) or members of the organic anion transporting polypeptide (OATP) family [51]. These properties confer upon CGA substantial therapeutic potential for central nervous system disorders (Figure 2).

## 6. Potential Mechanisms of CGA in the Treatment of Cognitive Impairment

### 6.1. Neuroprotective Effects

Chlorogenic acid (CGA) exerts neuroprotective effects through multiple synergistic mechanisms, thereby ameliorating cognitive impairment. These mechanisms operate at several levels by maintaining neuronal survival, regulating neurotransmitter systems, modulating the immune microenvironment and improving energy metabolism. First, CGA treatment effectively preserves the number of surviving neurons in the prefrontal cortex, hippocampal CA1, and CA3 regions [52] and prevents the depletion of brain-derived neurotrophic factor (BDNF) in these areas [52]. The stabilization of BDNF contributes to the maintenance of long-term potentiation of synaptic transmission, thereby promoting neurogenesis, synaptic plasticity, and learning and memory capacity. In this context, CGA also regulates synapse-associated proteins: on one hand, it upregulates synapsin I expression in fetal mice raphe nucleus neurons, promoting serotonin (5-HT) release and consequently enhancing synaptic plasticity [53,54]; on the other hand, against neurotoxic proteins, CGA can inhibit the interaction between oxidized dopamine and α-synuclein (α-syn) in vitro, thereby preventing the aberrant aggregation of α-syn and mitigating the risk of dopaminergic neuronal death [55].

**Figure 3:**
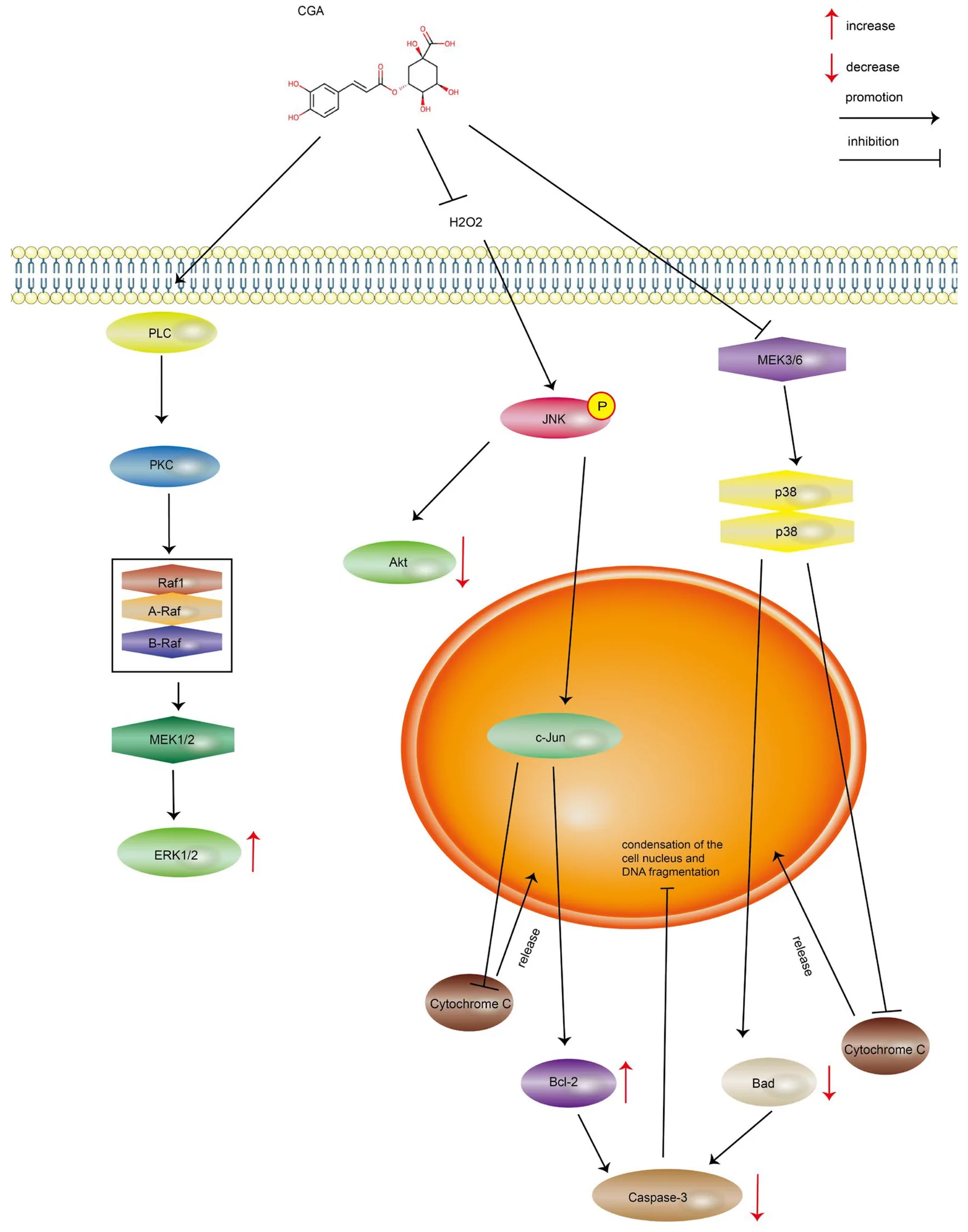
PKC, p38 MAPK, and JNK/c-Jun signaling pathways. Abbreviations: c-Jun N-terminal kinase (JNK), members of the mitogen-activated protein kinase (MAPK). Biological models: primary cultures of rat cortical neurons obtained from the cortical lobes of E18 Sprague-Dawley rat embryos, exposed to L-glutamate-induced excitotoxicity/oxidative stress; TMT-induced neurobehavioral dysfunctions in male C57BL/6J mice and in vitro oxidative neuronal cell death model using PC12 rat pheochromocytoma cells exposed to H_2_O. Symbols and abbreviations: The red upward arrow indicates an increase (upregulation) in expression or activity; the red downward arrow indicates a decrease (downregulation); the black horizontal arrow (→) denotes promotion or activation; the black line with a flat end (⊣) denotes inhibition or suppression. Image adapted from Servier Medical Art (https://smart.servier.com), licensed under CC BY 4.0 (https://creativecommons.org/licenses/by/4.0/ (accessed on 16 September 2026)).

Second, CGA acts on multiple neurotransmitter systems. In the dopaminergic system, tyrosine hydroxylase (TH) is the rate-limiting enzyme of dopamine biosynthesis [56]. CGA enhances TH expression and protects dopaminergic neurons [57], suggesting that CGA may promote TH-mediated dopamine biosynthesisand consequently regulating neural activities related to memory, emotion, and cognition [58,59]. In the cholinergic system, CGA ameliorates trimethyltin chloride (TMT)-induced acetylcholine disturbances [21], thereby elevating synaptic acetylcholine levels and promoting neural plasticity and network connectivity [60]. Furthermore, CGA regulates intracellular calcium homeostasis, alleviating the abnormal intracellular Ca^2+^ elevation induced by glutamate overstimulation and counteracting excitotoxic injury. This protective effect is closely associated with the PKC signaling pathway (Figure 3) [61].

Finally, CGA provides systemic support for neuroprotection by regulating the immune microenvironment and energy metabolism. In terms of immune regulation, the expression of anti-inflammatory cytokines (IL-4, IL-13) is enhanced by CGA, whereas the release of pro-inflammatory cytokines (TNF-α, IL-6, IL-1β, IL-2) is suppressed [22,62], thereby attenuating neuroinflammation. Of particular importance, CGA also improves systemic energy metabolism: indirect evidence from an in vitro HepG2 study, although not in a cognitive or neuronal model, indicates that it upregulates the activity of glycolysis, the tricarboxylic acid cycle (TCA) cycle, and the mitochondrial respiratory chain, promoting ATP synthase expression [63]. This finding supports, but does not directly prove, a neuronal mechanism of CGA. This metabolic modulation is also reflected in substrate profiles, with decreased plasma concentrations of glycolytic intermediates such as D-mannose and fructose-6-phosphate, alongside elevated phosphocholine levels, indicating active membrane phospholipid metabolism and an overall improvement in the energy supply efficiency of neural cells [64].

### 6.2. Regulation of the Gut–Brain Axis

The “gut–microbiota–brain axis” is a two-way communication network linking the gut and the brain. Its regulatory mechanisms encompass multiple dimensions, including signaling by microbial metabolites (e.g., short-chain fatty acids [SCFAs]), preservation of intestinal barrier integrity, and cascade regulation of central neuroinflammatory responses. SCFAs not only modulate synaptic plasticity and neurotransmitter levels but also influence neuroinflammatory and neurodegenerative processes [64]. Within this axis, the dynamic balance of the gut microbiota, particularly the increase and decrease in beneficial bacteria that produce SCFAs (such as those in the Clostridiales family) and opportunistic pathogens (such as Helicobacter), exerts significant regulatory effects on cognitive function by influencing the systemic inflammatory state [21,64].

**Figure 4:**
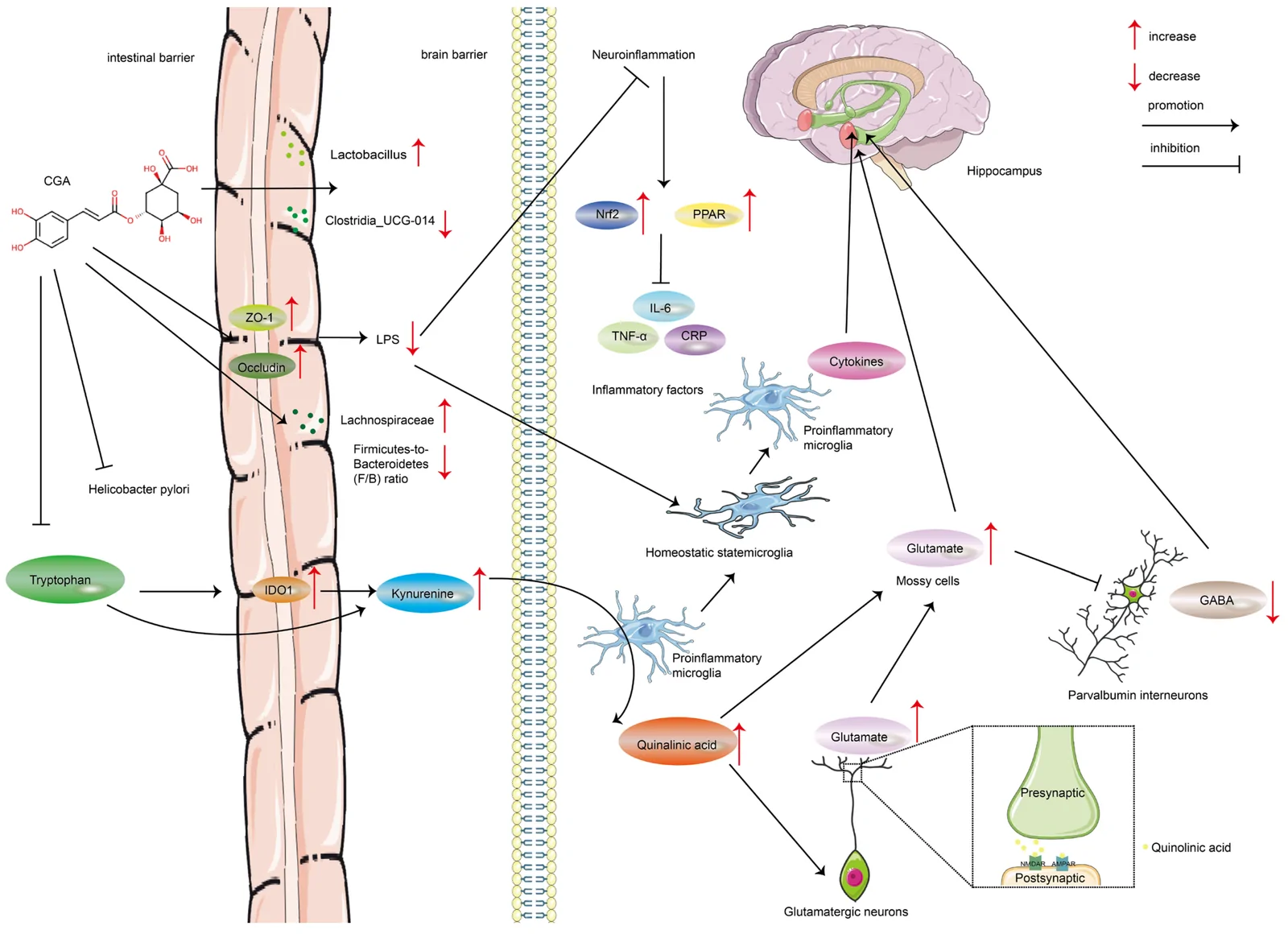
Tryptophan metabolic pathway and Nrf2/PPAR signaling pathway. Biological models: male C57BL/6 mice (8 weeks old, SPF), sleep deprivation (SD) for 48 h. Symbols and abbreviations: The red upward arrow indicates an increase (upregulation) in ex-pression or activity; the red downward arrow indicates a decrease (downregulation); the black horizontal arrow (→) denotes promotion or activation; the black line with a flat end (⊣) denotes inhibition or suppression. Image adapted from Servier Medical Art (https://smart.servier.com), licensed under CC BY 4.0 (https://creativecommons.org/licenses/by/4.0/ (accessed on 16 September 2026)).

Emerging evidence suggests that CGA, administered via oral gavage, may improve gut microecology from both microbiota structural and metabolic dimensions, and may thereby exert cognitive protective effects through the gut-brain axis. Regarding microbiota structure, whether CGA intervention specifically increases the abundance of beneficial Lachnospiraceae remains to be directly demonstrated; however, Lachnospiraceae, including Lachnospira eligens, have been implicated in epileptogenesis [65]. In addition, it suppresses the proliferation of the pathogenic Helicobacter, and effectively reduces the Firmicutes-to-Bacteroidetes (F/B) ratio, thereby restoring gut microbial homeostasis [64]. And this structural optimization directly alleviates colon inflammation, thereby contributing to the restoration of the colonic immune microenvironment [66]. In the general literature on microbiota metabolism, CGA has been reported to significantly elevates fecal levels of acetate, propionate, and butyrate (SCFAs) in mice [67]. These SCFAs not only contribute to the remodeling of gut microbial equilibrium and the maintenance of barrier integrity but also activate intestinal stem cells and promote epithelial regeneration [42,68,69], providing a sustained driving force for intestinal barrier repair. Another indirect evidence shows that CGA also regulates the tryptophan metabolic pathway through gut microbiota modulation [70], effectively inhibiting the accumulation of the harmful metabolite kynurenine in the mouse hippocampus [19], thereby averting its associated neurotoxicity, excitotoxicity, and neuroinflammatory responses. Furthermore, CGA can ameliorate hippocampus-dependent spatial memory-related immune profiles through the Nrf2/PPAR signaling pathway [71] (Figure 4).

### 6.3. Inhibition of Neuroinflammation

Neuroinflammation refers to a series of immune responses mediated by neuroimmune cells within the central nervous system, playing a crucial role in the development of cognitive dysfunction. The polarization state of microglia is considered the core link in regulating the inflammatory process. Under pathological conditions, resting microglia become activated and polarize into pro-inflammatory (M1) and anti-inflammatory (M2) phenotypes. M1 microglia secrete pro-inflammatory cytokines capable of damaging neuronal cells, whereas M2 microglia suppress inflammation and exert neuroprotective effects. Therefore, shifting microglia towards the M2 phenotype has been recognized as a critical therapeutic strategy for neuroinflammatory conditions. Studies have shown that CGA can inhibit BV-2 microglial polarization toward the M1 phenotype in vitro and promote M2 polarization, thereby attenuating neuroinflammation and ameliorating cognitive dysfunction [72].

However, the regulatory network of neuroinflammation extends far beyond intrinsic phenotypic switching of microglia. During the inflammatory cascade, activated microglia release various chemokines that recruit peripheral immune cells (e.g., neutrophils) into the central nervous system [73]. Further research has demonstrated that human neutrophil elastase (HNE), released by neutrophils, acts as a key inflammatory protease [74,75] that may indirectly contribute to microglia, promote pro-inflammatory cytokine release, and damage neuronal and synaptic function, thereby exacerbating cognitive impairment. Consequently, inhibiting HNE activity represents another potential intervention target. Notably, CGA has been confirmed to effectively inhibit HNE activity [76].

**Figure 5:**
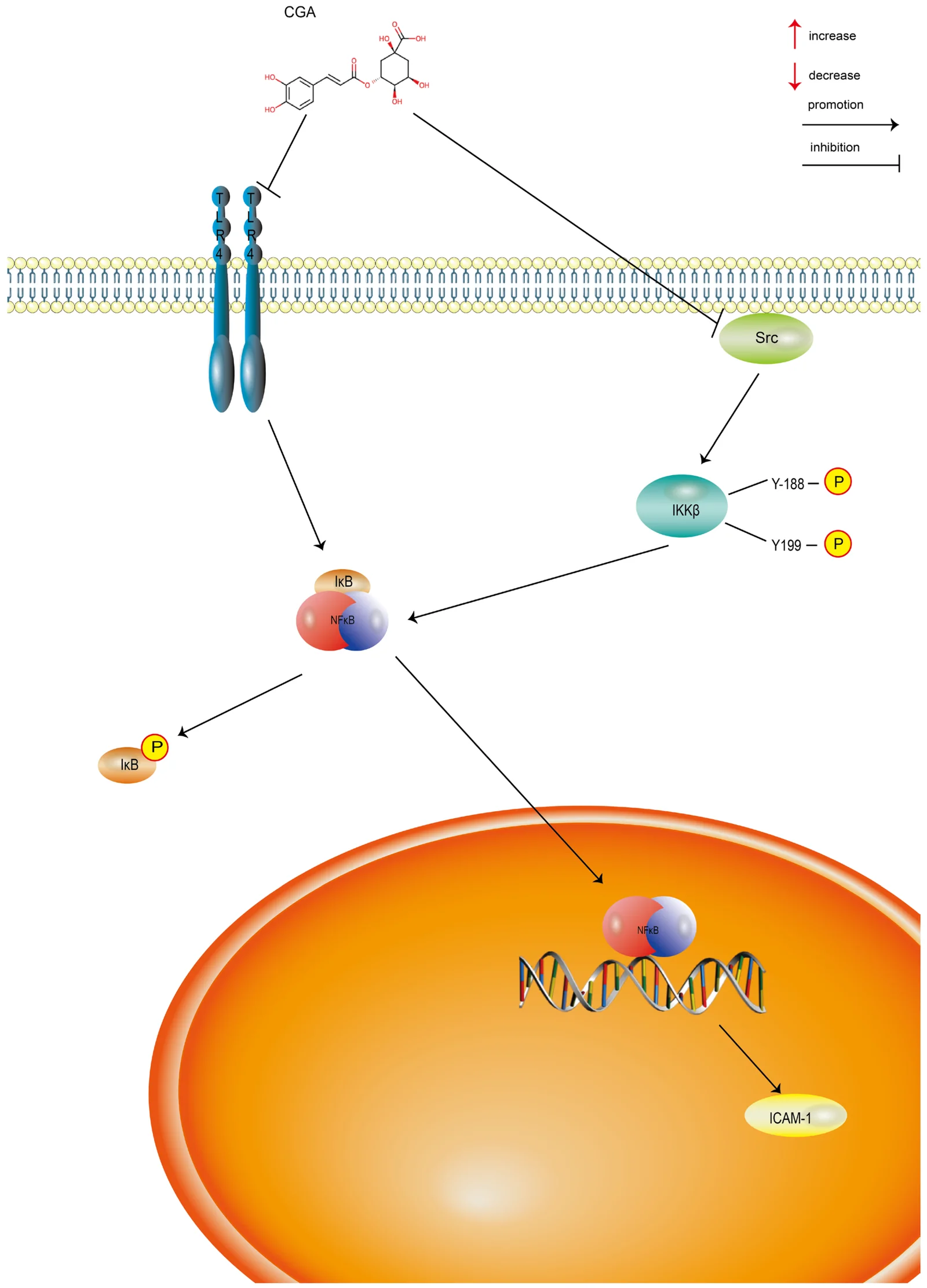
TLR4/NF-κB signaling pathway. Abbreviations: Toll-like receptor 4 (TLR4). Symbols and abbreviations: The red upward arrow indicates an increase (upregulation) in expression or activity; the red downward arrow indicates a decrease (downregulation); the black horizontal arrow (→) denotes promotion or activation; the black line with a flat end (⊣) denotes inhibition or suppression. Image adapted from Servier Medical Art (https://smart.servier.com), licensed under CC BY 4.0 (https://creativecommons.org/licenses/by/4.0/ (accessed on 16 September 2026)).

In terms of inflammatory signal transduction, the Toll-like receptor 4 (TLR4)/nuclear factor-κB (NF-κB) signaling pathway is a recognized key inflammatory cascade. Activation of this pathway releases large quantities of inflammatory cytokines, disrupts blood–brain barrier and cerebral white matter integrity, and eventually leads to cognitive impairment [77]. CGA-enriched coffee can downregulate the TLR4/NF-κB pathway, attenuating trimethyltin chloride (TMT)-induced neuroinflammation [21], and inhibiting the excessive activation of astrocytes and microglia. Additionally, SRC, an upstream activator of NF-κB, also participates in inflammatory signal transduction and is closely associated with cognitive deficits [78,79,80]. CGA in chokeberry and bilberry fruit extracts may have been demonstrated to exert a potent inhibitory effect on Src activity in prior research [81]. (Figure 5). However, this remains to be demonstrated using isolated CGA in neuronal or cognitive models. It is noteworthy that the neuroinflammatory attenuation by CGA may involve broader systemic immune regulation, as it can significantly affect pathways related to immune diseases (ko05322) and the immune system (ko04610) [71].

### 6.4. Inhibition of Oxidative Stress Damage

Oxidative stress drives neuronal vulnerability and disrupts the basal metabolic homeostasis essential for neuronal survival, thereby accelerating the pathological process of neurodegenerative diseases and ultimately leading to progressive cognitive decline [82]. CGA, by virtue of its distinctive phenolic hydroxyl structure, possesses multi-layered antioxidant capabilities and has been demonstrated to effectively mitigate oxidative damage across various pathological models [83].

In terms of direct free radical scavenging, the phenolic hydroxyl groups in the CGA molecule can rapidly react with free radicals, donating hydrogen atoms to neutralize hydroxyl radicals and superoxide anions, thereby suppressing their activity [84]. CGA can also form protein-polyphenol complexes through hydrogen bonding, thickening the interfacial layer and limiting free radical diffusion, thus enhancing the antioxidant capacity of proteins [83]. Moreover, CGA exhibits DPPH radical scavenging activity [85], and can both scavenge oxidizing radicals and repair pre-formed oxidizing radical adducts of purine nucleotides through fast electron transfer [86]. In peroxynitrite-related pathological processes, CGA has also been reported to possess scavenging capacity, potentially conferring protective effects in immature brain ischemia models [87,88].

At the level of oxidative enzyme activity regulation, CGA can downregulate the expression of NADPH oxidase (NOX) and dual oxidase 2 (DUOX2), inhibiting mitochondrial reactive oxygen species (ROS) production [89]. Simultaneously, CGA can bind non-covalently to the flavin adenine dinucleotide (FAD) cofactor site of xanthine oxidase, interfering with the electron transfer process and thereby inhibiting the xanthine oxidation reaction [90,91].

Regarding the endogenous antioxidant system, existing studies indicate that CGA can upregulate the expression of copper/zinc superoxide dismutase (SOD1) and manganese superoxide dismutase (SOD2), reverse the decline in superoxide dismutase (SOD) and catalase (CAT) activities caused by cerebral ischemia or glutamate exposure, and increase the levels of malondialdehyde (MDA), the terminal product of lipid peroxidation [22,62,66,92]. Additionally, CGA also modulates the expression of thioredoxin, further participating in the ROS clearance process [93].

**Figure 6:**
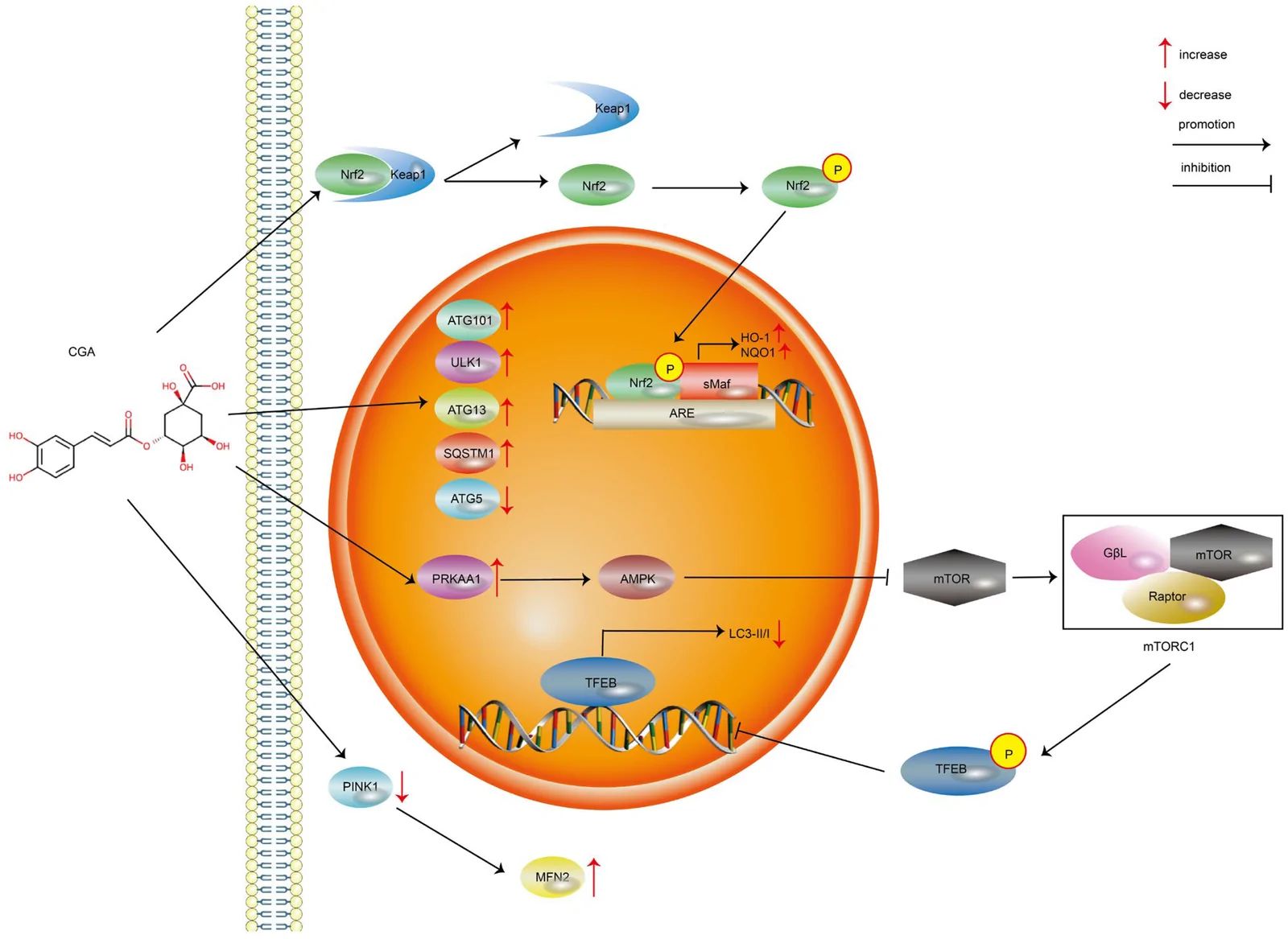
Nrf2/ARE and mTOR/TFEB signaling pathways. Abbreviations: mechanistic target of rapamycin (mTOR), Transcription factor EB (TFEB), mitofusin 2 (MFN2). Biological models: differentiated human neuroblastoma SHSY5Y cells and aged male C57BL/6 mice induced by ISO. Symbols and abbreviations: The red upward arrow indicates an increase (upregulation) in expression or activity; the red downward arrow indicates a decrease (downregulation); the black horizontal arrow (→) denotes promotion or activation; the black line with a flat end (⊣) denotes inhibition or suppression. Image adapted from Servier Medical Art (https://smart.servier.com), licensed under CC BY 4.0 (https://creativecommons.org/licenses/by/4.0/ (accessed on 16 September 2026)).

At the signaling pathway level, CGA can activate the Nrf2/ARE signaling pathway, promoting Nrf2 nuclear translocation and increasing the expression of its downstream targets heme oxygenase-1 (HO-1) and NAD(P)H quinone dehydrogenase 1 (NQO1), thereby protecting against isoflurane (ISO)-induced cognitive impairment [22] (Figure 6).

Furthermore, CGA can display indirect antioxidant effects via the regulation of metal ions. It can reduce Fe^3+^ to Fe^2+^ [85], and inhibit Fe^2+^ autoxidation [94].

### 6.5. Promotion of Autophagy

Autophagy is a cellular mechanism wherein lysosomes are employed to break down and recycle damaged organelles and macromolecules. Its functional homeostasis is critical for maintaining the normal physiological activities of neurons. Dysregulation of the autophagy–lysosome pathway has been identified as an important pathological mechanism of cognitive dysfunction. In this pathway, p62/SQSTM1, as a multifunctional scaffold protein, mediates the targeted delivery of ubiquitinated protein aggregates to autophagosomes by binding microtubule-associated protein 1 light chain 3 (LC3). The accumulation of p62 is commonly regarded as a direct marker of blocked autophagic flux [95]. Furthermore, pathological changes such as amyloid-β (Aβ_25–35_) deposition and hyperphosphorylation and aggregation of Tau protein can both induce autophagy and, conversely, be exacerbated by impaired autophagic clearance.

The activity of the autophagy-lysosome pathway is finely modulated by multiple signaling molecules. Mammalian target of rapamycin (mTOR), a highly conserved serine/threonine kinase predominantly localized to the lysosomal membrane, is a key negative regulator of autophagy initiation. Transcription factor EB (TFEB), as the master regulator of lysosomal biogenesis, is phosphorylated by mTORC1 at multiple serine residues, including Ser211. Phosphorylation at Ser211 promotes TFEB association with 14-3-3 proteins and its retention in the cytoplasm [96]. Inhibition of mTORC1 results in TFEB dephosphorylation and nuclear translocation, thereby activating the autophagy-lysosome pathway. Cathepsin D, the most abundant aspartyl protease in lysosomes, is activated only under acidic conditions and is responsible for degrading intracellular misfolded or aggregated proteins, playing a core role in the terminal degradation phase of the autophagy-lysosome pathway [97].

Targeting the aforementioned regulatory network, the study by Lijuan Gao et al. revealed the neuroprotective mechanism of CGA. They found that CGA treatment restored autophagic flux homeostasis in the brains of APP/PS1 Alzheimer’s disease model mice by inhibiting the mTOR/TFEB signaling pathway [97], thereby ameliorating cognitive impairment. Specifically, CGA significantly inhibited the excessive autophagosome formation induced by Aβ_25–35_ in SH-SY5Y cells, enhanced lysosomal acidification and degradative activity, and upregulated cathepsin D protein expression [97,98]. In terms of autophagic flux dynamics, the LC3-II/I ratio reflects the steady-state abundance of autophagosomes rather than flux per se. CGA treatment decreased the LC3-II/I ratio and lowered p62 protein levels, and this was accompanied by increased mCherry^+^GFP^−^ puncta, enhanced lysosomal function, and CQ-sensitive protection, suggesting effective clearance of accumulated autophagosomes and restoration of impaired autophagic flux [98].

Notably, transcriptional dysregulation of autophagy-related genes (ATG101, ATG13, ULK1, SQSTM1/p62, and ATG5) can lead to defective autophagosome formation, impaired lysosomal degradation, and interrupted mitophagy. Among these, autophagosome formation depends on the ATG5 protein, whose reduced expression inhibits autophagy. Mitophagy is primarily mediated by PTEN-induced putative kinase 1 (PINK1), which tags damaged mitochondria for selective clearance, while mitofusin 2 (MFN2) maintains mitochondrial network integrity by mediating outer mitochondrial membrane fusion. AMP-activated protein kinase (AMPK, encoded by the PRKAA1 gene), as a cellular energy sensor, can initiate autophagy by inhibiting mTOR activity. The research by Apoorv Sharma et al. further elucidated the mechanism of CGA at the gene expression level: CGA reduced PINK1 expression, thereby alleviating Aβ_1–42_-induced mitophagy, and upregulated MFN2 to restore mitochondrial fusion. At the autophagy pathway level, following CGA treatment, PRKAA1 (AMPK) mRNA levels were elevated, MTOR mRNA levels were decreased, and the transcriptional expression of ATG101, ATG13, ULK1, and SQSTM1 (p62) was increased, while ATG5 levels exhibited a declining trend [95] (Figure 6).

### 6.6. Inhibition of Apoptosis

CGA can ameliorate cognitive function by inhibiting neuronal apoptosis, and its protective effects have been validated in both oxidative stress and excitotoxicity models. At the mechanistic level, CGA primarily targets members of the mitogen-activated protein kinase (MAPK) family: it can inhibit the aberrant phosphorylation of c-Jun N-terminal kinase (JNK) and p38 MAPK induced by hydrogen peroxide (H_2_O_2_), thereby blocking the initiation of apoptotic signaling [99]. More importantly, CGA exhibits bidirectional regulatory characteristics—while blocking death signals, it can also, as suggested by indirect evidence, dose-dependently reverse the downregulation of ERK1/2 and Akt protein expression, thereby reactivating cell survival signaling [100]. The combined actions of these two pathways ultimately reverse key events in the mitochondrial apoptotic pathway, including upregulation of the anti-apoptotic protein Bcl-2, downregulation of the pro-apoptotic protein Bad, and inhibition of cytochrome c release into the cytoplasm [22,100]. Ultimately, these changes preserve pro-caspase-3 and suppress its cleavage into active caspase-3, thereby diminishing neuronal nuclear condensation and DNA fragmentation [99] (Figure 3).

In in vivo studies, Yu Xi et al. found that oral CGA administration can reverse neuronal damage in the mouse hippocampal CA1 region and dentate gyrus (DG) via the JNK/c-Jun pathway, specifically manifesting as reduced eosinophilic cytoplasm, inhibited nuclear pyknosis and karyolysis, and abundant Nissl substance in surviving neurons [21]. Notably, aberrant activation of the JNK/c-Jun pathway is strongly linked to heightened neuronal apoptosis and is regarded as one of the key pathological causes of neurodegenerative diseases [21], providing an important theoretical basis for the intervention value of CGA (Figure 3).

### 6.7. Inhibition of Amyloid-β Formation

Amyloid-β (Aβ) is produced through sequential proteolytic processing of amyloid precursor protein (APP) by β-secretase (primarily β-site APP cleaving enzyme 1, BACE1) and γ-secretase. Under physiological conditions, the synthesis and removal of Aβ exist in dynamic equilibrium; however, when brain extracellular Aβ levels become abnormally elevated, they significantly inhibit hippocampal long-term potentiation (LTP), thereby constituting one of the key initiating factors of memory impairment [101]. These excessively accumulated Aβ monomers have a high propensity for self-assembly, forming soluble oligomers—the most neurotoxic species—which further reassemble into protofibrils and ultimately deposit as insoluble amyloid plaques [102]. More importantly, excessive Aβ accumulation not only directly induces synaptic toxicity and neurotoxicity but also drives tau phosphorylation and aggregation, promoting the generation of neurofibrillary tangles, eventually leading to cognitive impairment [103].

Targeting the aforementioned pathological cascade, multiple in vivo studies in recent years have revealed the multi-faceted intervention potential of coffee polyphenols, isolated 5-CQA, and solanesol-CGA nanomicelle formulations in anti-Alzheimer’s therapy. Current evidence indicates that these agents can act on both the downstream stages of Aβ aggregation and its clearance pathways. Specifically, evidence obtained with coffee polyphenols has shown that these polyphenols can directly degrade pre-formed Aβ protofibrils and promote their disaggregation [104]. Evidence obtained with isolated 5-CQA has shown that this compound modulates brain Aβ clearance mechanisms, significantly reducing Aβ deposition and plaque burden in APP/PS2 transgenic mouse models, while effectively improving cognitive behavior and attenuating neuronal loss [105]. In addition, Chenchen Wang et al. further employed nano-delivery technology to construct CI@SPK nanomicelles based on solanesol and CGA; this solanesol-CGA nanomicelle formulation not only reduced Aβ plaque burden in APP/PS1 mice but also significantly inhibited neuroinflammatory responses, thereby synergistically ameliorating learning and memory deficits [106]. The evidence from in vivo and in vitro studies on these mechanisms has been systematically summarized in Table 2.

## 7. Human Evidence and Translational Limitations

Although in vitro and in vivo experimental evidence is encouraging, clinical evidence that chlorogenic acid (CGA) improves cognitive function remains limited. Among the human randomized controlled trials (RCTs) relevant to CGA and cognition from which specific trial details could be extracted, the randomized, double-blind, placebo-controlled trial by Saitou et al. is the most directly informative [107]. This trial assigned 38 healthy participants to either a CGA-added beverage group or a placebo group for 16 weeks, and cognitive function was assessed using the Japanese version of CNS Vital Signs (Cognitrax) [107]. The CGA group showed significantly greater increases in Cognitrax domain scores for motor speed, psychomotor speed, and executive function than the placebo group, together with improved shifting attention test scores; blood analyses also showed increased levels of apolipoprotein A1 and transthyretin, which are considered candidate biomarkers of early cognitive decline [107]. However, this trial has clear limitations: a small sample size; healthy participants rather than patients with diagnosed cognitive impairment or dementia; a short intervention period of only 16 weeks; and use of a CGA-added beverage rather than a purified CGA supplement, making generalization to other formulations and populations difficult [107]. More importantly, the trial did not report the absolute daily CGA dose, did not measure circulating CGA or its metabolite concentrations, and did not assess brain exposure [107]. Therefore, even though cognitive-related endpoints improved, the dose–response relationship between intake, circulating exposure, and central exposure cannot be determined.

Apart from the above trial, human CGA RCTs with extractable sample sizes, formulations, doses, and cognitive endpoints remain scarce; no studies were identified that directly compared CGA concentrations used in cell experiments with achievable human circulating or brain exposure. The currently extractable preclinical evidence is mainly from animal dosing studies. For example, in a gerbil model of transient forebrain ischemia, pretreatment with 30 mg/kg CGA attenuated cognitive impairment and exerted neuroprotective effects [62]; in a zebrafish model of hyperglycemia combined with chronic stress, 50, 100, or 200 mg/kg CGA modulated antioxidant defense and improved indices related to brain homeostasis [108]; and in scopolamine-induced memory-deficit mice, co-administration of CGA with cilomilast reversed cognitive deficits and reduced amyloid beta accumulation and neuroinflammatory markers [109]. The doses used in these animal studies cannot be directly converted to the human exposure that was not reported in the RCT, and bridging data among cellular concentrations, human circulating concentrations, and brain tissue concentrations are lacking.

In addition, formulation differences (purified supplements versus coffee or food matrices) have rarely been compared in the available evidence, and the only RCT from which details could be extracted used a CGA-added beverage, further limiting extrapolation [107]. Doses used in experimental studies are generally much higher than those achievable through habitual dietary intake; therefore, it remains unclear whether the preclinical effects are relevant to habitual dietary consumption [62,108,109]. Safety data for long-term high-dose supplementation also remain scarce.

Therefore, these limitations should be carefully considered when interpreting the current evidence. Future studies should include larger and more diverse populations, clearly report CGA dose, formulation, and intervention duration, directly measure circulating metabolites and brain exposure, establish quantitative comparisons between preclinical concentrations/doses and human achievable exposure, and systematically evaluate the safety of long-term high-dose supplementation [107].

## 8. Conclusions

This review summarizes the potential mechanisms by which CGA may ameliorate cognitive impairment. At present, the evidence supporting these mechanisms is predominantly preclinical. Preclinical studies suggest that CGA may exert protective effects via multiple pathways, including neuroprotection, modulation of gut microbiota composition and metabolites, suppression of neuroinflammation, reduction of oxidative stress injury, regulation of autophagy and apoptosis, and inhibition of Aβ formation. The implicated signaling pathways include the JNK/c-Jun, TLR4/NF-κB, Nrf2/PPAR, Nrf2/ARE, and mTOR/TFEB pathways, among others. Although the proposed mechanisms of action of CGA are broad, encompassing diverse pathways and targets, clinical research on CGA in patients with cognitive impairment remains limited. There is also a lack of comprehensive studies on the safety, toxicity, teratogenicity, and carcinogenicity of CGA. Furthermore, the mechanisms underlying certain pathways still require further investigation. Additional preclinical and clinical studies are needed to determine whether CGA is therapeutically effective in patients with cognitive impairment.

## Figures and Tables

**Table 1:** CGA content in common dietary and medicinal sources.

Name	Plant Part	FW/DW Basis	Extraction Conditions	Analytical Method	Content (μg/g)	Reference
**sweet potato**	leaves	DW	0.5 g leaf powder + 5 mL methanol; stir 25 °C, 2 h; centrifuge 4 °C, 12,000 rpm, 10 min; re-extract twice; combine, filter, concentrate to dryness; SPE cleanup	UPLC-ESI-MS/MS (Waters, Milford, MA, USA)	9–15	[25]
**potato**	tubers	FW	Freeze-dried; defatted with hexane (Soxhlet, 16 h); refluxed with 20 mL ethanol (6 h); filtered (0.45 μm)	UV spectrophotometry (Perkin-Elmer)	9700–18,700	[26]
**o** **riental pear**	whole fruit	FW	95% EtOH (−18 °C) + 0.4% H_3_PO_4_; ultrasonic 5 min; centrifuge; evaporate; dissolve in water; 0.45 µm filter	HPLC-PDA and LC/MS (Agilent Technologies, Palo Alto, CA, USA)	163	[27]
**apple**	core	DW	Freeze-dried tissue (0.5 g) extracted with 20 mL methanol/10% acetic acid (85:15) in ultrasonic bath 30 min; concentrated at 35 °C; reconstituted; diluted to 25 mL; filtered 0.2 µm	RP-HPLC	2100	[28]
**apple**	seeds	DW	Freeze-dried tissue (0.5 g) extracted with 20 mL methanol/10% acetic acid (85:15) in ultrasonic bath 30 min; concentrated at 35 °C; reconstituted; diluted to 25 mL; filtered 0.2 µm	RP-HPLC	1100	[28]
**apple**	fles	DW	Freeze-dried tissue (0.5 g) extracted with 20 mL methanol/10% acetic acid (85:15) in ultrasonic bath 30 min; concentrated at 35 °C; reconstituted; diluted to 25 mL; filtered 0.2 µm	RP-HPLC	480	[28]
**coffee**	grounds	DW	Various methods (water, MeOH, EtOH, acetone, DES; SLE/UAE/MAE/SFE; 20–150 °C, 1–150 min)	HPLC	350–85,620	[29]
***Lonicera japonica*** **(Jinyinhua)**	flower	DW	MAE: 60% ethanol, 200 W, 28 min, 1 g/100 mL	DART-MS/MS (Thermo Scientific, San Jose, CA, USA and IonSense, Saugus, MA, USA); HPLC-UV (Waters Corporation, Milford, MA, USA)	21,810–32,610	[30]
***Chrysanthemum morifolium*** **(Juhua)**	flower	DW	NR	HPLC (Agilent Technologies, Santa Clara, CA, USA)	4936.7–5498.4	[31]
***Phellodendron amurense*** **(Huangbo)**	leaves	DW	0.2 g; 15 mL 70% methanol; shake 30 min; centrifuge; filter 0.20 μm; no hydrolysis	UHPLC-PDA (Waters, MA, USA)	400–17,250	[32]
***Cichorium intybus root*** **(Jujuigen)**	root	DW	H2O/CHCl3/MeOH (30/30/40, *v*/*v*/*v*); 100 mg powder + 1.5 mL solvent; 17 h dark, room temperature; centrifugation/filtration	HPLC-DAD (Shimadzu, Marne la Vallée, France)	100.2	[33]
***Bretschneidera sinensis*** **(Bolaishu)**	leaves	DW	2.0 g dried powder; Soxhlet with chloroform 20 h; then boiling methanol (5 × 100 mL); evaporated; redissolved in 25 mL MeOH	RP-HPLC (Palo Alto, CA, USA; Cotati, CA, USA; Lafayette, CA, USA; Darmstadt, Germany)	1152–44,500	[34]
***Bretschneidera sinensis*** **(Bolaishu)**	underground parts	DW	2.0 g dried powder; Soxhlet with chloroform 20 h; then boiling methanol (5 × 100 mL); evaporated; redissolved in 10 mL MeOH	RP-HPLC (Palo Alto, CA, USA; Cotati, CA, USA; Lafayette, CA, USA; Darmstadt, Germany)	1050–4610	[34]
***Foeniculum vulgare*** **(Huixiang)**	NR	DW	250 mg dried sample + 25 mL 20% methanol, ultrasonic 30 min, room temp; filtered 0.45 µm	HPLC (Thermo Electron Corporation, San Jose, CA, USA)	614–2325	[35]
***Eucommia ulmoides*** **(Duzhong)**	three parts (leaves, barks and fruits)	DW	0.5 g powder + 25 mL methanol; ultrasonic 30 min (250 W, 20 kHz); filtered through 0.45 μm	HPLC-DAD (Agilent, USA)	980.08–7375.79	[36]
***Schisandra chinensis*** **(Wuweizi)**	fruits	DW	0.5 g DW + 50 mL methanol, reflux 2 h	HPLC-DAD (Merck Hitachi)	45.5	[37]
***Acanthopanax senticosus*** **(Ciwujia)**	NR	DW	1.0 g powder + 40 mL ethanol, ultrasonic 30 min; filtered, evaporated, redissolved in 10 mL methanol; 20 μL injection	HPLC-DAD	89.56–643.9	[38]
***Aralia nudicaulis*** **L.**	rhizomes	DW	Dried rhizomes were defatted with hexane, refluxed with MeOH (3 × 1.5 h), partitioned with EtOAc, and purified on HP-20 Diaion (MeOH: H2O, 0 → 100%); 50% MeOH eluate = FEPC. Quantification: 0.5 g powder + 10 mL 70% MeOH, sonication 15 min × 3, centrifuga-tion, evaporation, re-dissolution in 2 mL MeOH	HPLC-UV 320 nm; LC-MS (negative ESI/APCI) (Agilent Technologies, Mississauga, ON, Canada)	156,000	[39]
***Sanghuangporus baumii*** **(Pilát)**	fruiting bodies	DW	0.1 g powder + 1.5 mL 70% methanol–water (*v*/*v*); vortex 5 min; ultrasonic 30 min; centrifuge 12,000 rpm, 20 min, 4 °C; filter 0.22 μm	UHPLC-QqQ (SCIEX, Framingham, MA, USA)	10,000–80,000	[40]

FW, fresh weight; DW, dry weight; NR, not reported in the original table or cited reference.

**Table 2:** Summary of mechanistic evidence for chlorogenic acid (CGA) in cognitive impairment.

Experimental Model and Cognitive Impairment Inducer	CGA Form and Purity Dose or Concentration, Administration Route and Treatment Duration	Principal Outcomes	Pathway	Reference
In vivo: sporadic Alzheimer’s Disease (SAD) model in adult male albino Swiss mice, induced by intracerebroventricular streptozotocin (ICV-STZ)	Obtained from SIGMA-USA; 5 mg/kg; oral; 26 days	Preserves the number of surviving neurons in the prefrontal cortex, hippocampal CA1, and CA3 regions and prevents the depletion of BDNF in these areas	no	[52]
In vitro: primary fetal rat raphe neurons, corticosterone (Cort, 10 μM) was used to induce neuronal injury	Obtained from National Institutes for Food and Drug Control (Beijing, China). CGA was applied with concentrations of 0.001, 0.01, 0.1, 1, 10 μM; 0.5–1.0 nM; 1 nM, 5, 7, 8 days	Upregulates synapsin I, promotes serotonin (5-HT) release, enhances synaptic plasticity	no	[54]
In vitro: α-syn overexpressing PC12 cells	Obtained from MP Biomedicals, LLC (Irvine, CA, USA). CGA was added to the cell culture medium, with an increasing concentration of 0–10 µM for 30 min	Inhibits the interaction between oxidized dopamine and α-syn	no	[55]
In vivo: 1-methyl-4-phenyl- 1,2,3,6-tetrahydropyridine (MPTP) -intoxicated mouse model of Parkinson’s disease	Obtained from Sigma-Aldrich (St. Louis, MO, USA).; 25, 50, and 100 mg/kg body weight; oral; once daily for 24 days	Enhances TH expression and protects dopaminergic neurons	no	[57]
In vivo: TMT-induced cognition impairment in male C57BL/6J mice	Obtained from TCI (Shanghai, China); 30 mg/kg; oral gavage; 11 days	Ameliorats acetylcholine disturbances; increases Lactobacillus johnsonii and L. murinus; attenuates neuroinflammation	JNK/c-Jun and TLR4/NF-kB pathways	[21]
In vitro: cortical neurons obtained from the cortical lobes of E18 Sprague-Dawley rat embryos, lglutamate plus glycine were used to induced glutamate-excitotoxicity	Purchased from Sigma Aldrich Spain (St. Louis, MO, USA).; dissolved in DMSO as a 100 mM stock solution; 100 μM, 2 h	Regulates intracellular calcium homeostasis, alleviates the abnormal intracellular Ca^2+^ elevation	PKC pathway	[61]
In vivo: isoflurane (ISO)-induced cognitive dysfunction in aged male C57BL/6 mice	Sigma-Aldrich, purity 95%; St. Louis, MO, USA; 30 mg/kg and 60 mg/kg; oral gavage; once daily for 7 days	Decreases IL-1β, TNF-α, IL-6, nuclear p65 NF-kB, cleaved caspase-3, and Bax, and increases IκBα and Bcl-2; increases SOD, CAT; promotes Nrf2 nuclear translocation and elevates HO-1 and NQO-1	Nrf2/ARE signaling pathway	[22]
In vivo: transient forebrain ischemia (TFI) cognitive impairment model in male Mongolian gerbils (6 months old); induced by ligating both common carotid arteries with non-traumatic aneurysm clips	7.5, 15, and 30 mg/kg CGA; intraperitoneal injection; once a day for 5 days	Reduces 4-HNE and DHE levels, increases SOD1 and SOD2, decreases TNF-α, IL-6, IL-1β, and IL-2; increased IL-4 and IL-13	no	[62]
In vivo: cognitive impairment male C57BL/6J mice model; induced by high-fat and high-fructose diet (HFFD).	obtained from Hunan World Wellbeing Bio-Tech Co., Ltd. (Hunan, China); purity > 98%; 150 mg/kg; Oral; 14 weeks	decreases D-mannose and fructose-6-phosphate, elevates phosphocholine levels, increases gut microbiota diversity and SCFA-producing bacteria	no	[64]
In vivo: cognitive impairment female ICR mice model; induced by D-galactose	Purchased from Solarbio (Beijing, China); purity ≥ 98%; 20 mg/kg/d; Oral gavage once daily; 8 weeks	Decreases colon IL-6 level	no	[66]
In vivo: cognitive impairments of male Kunming mice induced by PbCl2	Purchased from Sigma-Aldrich Chemical Company (St. Louis, MO, USA); purity ≥ 98%; 30 mg/kg/d; gavage administration; 8 weeks	Levates fecal levels of acetate, propionate, and butyrate (SCFAs)	no	[67]
In vivo: Lgr5-EGFR/Lgr5-EGFR-IRES-CreERT2 mice (8–12 weeks old), in vitro: intestinal organoids derived from these mice	Sigma, Shanghai, China; solution; 0.1 mmol/L; in vivo: received 14 days; in vitro: add into culture medium for 3 days.	Increases intestinal villous height and crypt depth, augments the area expansion and the number of budding intestinal organoids, upregulates Lgr5 and Olfm4, and promotes Ki67 and Edu	no	[69]
Memory deficits model of male C57BL/6 mice (8 weeks old, SPF); induced by sleep deprivation (SD) for 48 h	Obtained from Aladdin, Biotechnology Co., Ltd. (Shanghai, China); purity ≥ 98%; 30 and 60 mg/kg; oral gavage daily for a week	Decreases IL-6, TNF-α; increases the total blood cells (WBC), recovers lymphocyte population, inhibited platelet (PLT) hyperactivation; reduces ROS and iNOS; decreases Nrf2 and PPAR; increases zo-1 and occludin; increases villi height, decreases crypt depth; inhibited Clostridia_UCG-014; decreases ko05322 and ko04610	Nrf2/PPAR signaling pathway; pathways related to immune diseases (ko05322) and the immune system (ko04610)	[71]
In vivo: cognitive dysfunction of male C57BL/6 mice model; LPS-induced neuroinflammation; in vitro: LPS-stimulated BV-2 cell model	Purchased from Shanghai Yuanye Biotechnology Co., Ltd. (Shanghai, China); in vivo: 40 mg/kg/d, intraperitoneal injection, 11 days; in vitro: 50, 100, and 200 µM, added to cell culture; 24 h	Decreases TNF-, iNOS, CD86, increases Arg-1, IL-10, CD206	no	[72]
In vitro: M03-13 immortal human–human hybrid cell line	10, 25, 100 µM, add in to cell culture, 18 h; or 15 h	Downregulates the expression of NADPH oxidase (NOX) and dual oxidase 2 (DUOX2), inhibits mitochondrial reactive oxygen species (ROS) production	no	[89]
In vivo: alzheimer’s disease model of male ICR mice; induced by Scopolamine	Get from Sigma-Aldrich (St. Louis, MO, USA); in vivo: 0, 3, 6, or 9 mg/kg, oral, once; in vitro: 5, 25, 50, 100, 200, and 400 μg/mL; 30 min	Inhibits acetylcholinesterase activity and free radical scavenging activity	no	[92]
In vitro differentiated human neuroblastoma SHSY5Y cells induced by Aβ1-42	Purchased from Sisco Research Laboratories (Mumbai, India); 25 μM–200 μM; directly add to the cell culture dish; 24 h and 48 h	Elevates PRKAA1 (AMPK) mRNA levels, decreases MTOR mRNA levels, increases the transcriptional expression of ATG101, ATG13, ULK1, and SQSTM1 (p62), declines ATG5 levels,	no	[95]
In vivo: 3-month-old male APP/PS1 double transgenic mice; in vitro: human neuroblastoma SH-SY5Y cell line; in vitro injury inducer is Aβ25-35	Purity ≥ 98%, purchased from Baoji Herbest Bio-Tech Co., Ltd. (Hebei, China); in vivo: CGA 40 mg/kg. in vitro: CGA 3.125, 6.25, 12.5, 25, or 50 μM; In vivo: daily oral gavage; in vitro: added to the culture medium; in vivo: six months; in vitro: 24 h or 48 h	Reduces LC3B-II/LC3B-I and p62/SQSTM, beclin1 and Atg5; increases cathepsin D, decreases p-mTOR, increases nucleus TFEB	mTOR/TFEB signaling pathways	[97]
In vitro: H_2_O_2_-induced injury cell model using SH-SY5Y human neuroblastoma cells	5-caffeoylquinic acid; purity ≥ 98%, purchased from Baoji herbest Bio-Tech Co., Ltd.(Baoji, China); 6.25–50 µM; directly add to the cell culture dish; 24 h	Increases the expression of Bcl-2 and decreases the expression of Bax to inhibit cell apoptosis; inhibits the production of autophagosomes by down-regulating the expression of Beclin-1 and Atg5; increases the autophagic flux by reducing the levels of LC3-II/I and P62/SQSTM1; increase the nuclear level of TFEB and reduce the ratio of P-mTOR to mTOR	mTOR/TFEB signaling pathways	[98]
In vitro: PC12 cells were grown in DMEM supplemented induced by H_2_O_2_	Purchased from Sigma Chemical (St. Louis, MO, USA); 50 μL; directly add to the cell culture dish; 24 h	Prevents PARP cleavage, upregulates Bcl-XL and upregulates caspase-3, inhibited DNA fragmentation, blocks the accumulation of intracellular ROS, reduces the activation of JNK and p38 MAPK, inhibit the aberrant phosphorylation of c-Jun N-terminal kinase (JNK) and p38 MAPK	MAPK signaling pathway	[99]
In vivo: APP/PS2 double transgenic mouse model of Alzheimer’s disease overexpressing mutant human APP and presenilin-2	In vivo: coffee polyphenols (CPP) extract from green coffee beans, total polyphenol content 48.3%, containing 7.0% 3-CQA, 7.5% 4-CQA, 17.8% 5-CQA, 1.3% 3-FQA, 1.5% 4-FQA, 3.9% 5-FQA, 3.4% 3,4-diCQA, 2.4% 3,5-diCQA, and 3.5% 4,5-diCQA; In vitro: 5-CQA from Cayman Chemical; in vitro: fibrillization ThT measured every 5 min for 90 min; disaggregation incubated 7 days; Aβ aggregation 7–10 days.	Degrades pre-formed Aβ protofibrils and promotes their disaggregation	no	[104]
In vivo: Male APP/PS2 double-transgenic mice and wild-type littermates	Purchased from Cayman Chemical, Ann Arbor, MI, USA; 0.8% (*w*/*w*) 5-caffeoylquinic acid; oral; maintained on diets for 21–23 wk (6 mo); behavioral tests after 15–17 wk (4 mo); immunohistochemical and gene expression analyses after 6 mo	Inhibits the excessive production of Aβ, promote the degradation of Aβ, and enhances the clearance of Aβ, upregulates LRP1 and controlled perivascular AQP4 polarization, improves cognitive function	no	[105]
In vivo: seven-month-old male APP/PS1 AD mice; in vitro: three cell models (rat pheochromocytoma cells, PC12 cells, mouse microglia cells, BV2 cells and brain endothelial cells, bEnd.3 cells), induced by Aβ	Free chlorogenic acid (purchased from Macklin Biochemical Co., Ltd. (Shanghai, China)) is loaded onto CI@SPK; CHA 20 mg + Sol-PEG 20 mg + IR780 150 μg; in vivo: tail vein injection, 30 days; in vitro: Add the cell culture medium; Aβo/CI@SPK was co-cultured for 6 h, followed by NIR exposure for 5 min, and then continued for another 12 h.	reduces Aβ plaque burden, decreases TNF-α, IL-1β and IL-6 inhibits neuroinflammatory responses	no	[106]

## Data Availability

The original contributions presented in this study are included in the article. The data presented in this study are included in the article.

## Abbreviations

The following abbreviations are used in this manuscript:

CI	cognitive impairment
CGA	chlorogenic acid
Aβ	amyloid-β
TCMSP	Traditional Chinese Medicine Systems Pharmacology
BBB	blood–brain barrier
MCTs	monocarboxylate transporters
OATP	organic anion transporting polypeptide
BDNF	brain-derived neurotrophic factor
α-syn	α-synuclein
TMT	trimethyltin
TTR	transthyretin
TCA	tricarboxylic acid cycle
FAD	flavin adenine dinucleotide
SOD1	superoxide dismutase
SOD2	manganese superoxide dismutase
SOD	superoxide dismutase
CAT	catalase
MDA	malondialdehyde
HO-1	heme oxygenase-1
NQO1	NAD(P)H quinone dehydrogenase 1
ISO	isoflurane
LC3	light chain 3
SCFAs	short-chain fatty acids
F/B	Firmicutes-to-Bacteroidetes
*H. pylori*	*Helicobacter pylori*
M1	pro-inflammatory
M2	anti-inflammatory
HNE	human neutrophil elastase
HDC	histidine decarboxylase
TLR4	Toll-like receptor 4
NF-κB	nuclear factor-κB
NADPH	NOX oxidase
DUOX2	dual oxidase 2
ROS	reactive oxygen species
mTOR	mechanistic target of rapamycin
TFEB	Transcription factor EB
PINK1	putative kinase 1
MFN2	mitofusin 2
AMPK	AMP-activated protein kinase
MAPK	members of the mitogen-activated protein kinase
JNK	c-Jun N-terminal kinase
H_2_O_2_	hydrogen peroxide
DG	dentate gyrus
APP	amyloid precursor protein
